# Treatment with Anti-Interleukin 23 Antibody Ameliorates Disease in Lupus-Prone Mice

**DOI:** 10.1155/2013/861028

**Published:** 2013-06-06

**Authors:** Vasileios C. Kyttaris, Ourania Kampagianni, George C. Tsokos

**Affiliations:** ^1^Division of Rheumatology, Beth Israel Deaconess Medical Center, 330 Brookline Avenue, CLS-936, Boston, MA 02215, USA; ^2^Harvard Medical School, 25 Shattuck Street, Boston, MA 02115, USA

## Abstract

Interleukin 23 receptor expressing IL-17 producing T cells have been shown to be important in the development of murine lupus. The usefulness of IL-23 inhibition in ameliorating lupus nephritis is unknown. We hypothesized that inhibition of IL-23 will ameliorate nephritis in lupus-prone mice. To this end, we treated MRL/*lpr* lupus-prone mice for 6 weeks with a rat anti-IL-23p19 antibody, which resulted in delaying the onset of nephritis without affecting the production of anti-dsDNA antibodies. The effect of the treatment was hampered by the production of murine anti-rat IgG antibodies. The amelioration of murine lupus by IL-23 inhibition strengthens the rationale for targeting IL-23 in patients with systemic lupus erythematosus.

## 1. Introduction

Interleukin 23 (IL-23) is a member of the IL-12 family that is important for the generation and maintenance of Th17 cells. Th17 cells are defined by the production of the cytokine IL-17 and play an important role not only in the defense against microorganisms but also in autoimmune tissue damage. Generation of Th17 cells from naïve T cells depends on the cytokine milieu, namely, the presence of IL-6, IL-1*β*, TGF*β*, and IL-23. IL-23 in particular has been associated with the generation of a particularly proinflammatory subset of Th17 that expresses both IL-17 and IFN-*γ* [1]. The importance of IL-23 in the development of autoimmunity has been established by the fact that IL-23 receptor knockout does not develop experimental autoimmune encephalomyelitis (EAE) [2].

Systemic lupus erythematosus (SLE) is characterized by deficient T regulatory capacity, increased T : B cell cooperation as manifested by the production of T-cell-dependent high affinity IgG autoantibodies, and invasion of activated T cells into target tissues [3]. Several lines of evidence suggest that Th17 cells may play an important role in SLE and in particular lupus nephritis; for example, SLE T cells produce IL-17 spontaneously while IL-17+ T cells are found in the kidneys of SLE patients with nephritis. Similar to the case in patients with SLE, IL-17 expressing T lymphocytes are abundant in the spleen and kidneys of lupus-prone mice. Moreover, these cells express high levels of the IL-23 receptor with its expression increasing as the mice age and the disease progresses [4].

We have previously shown that lupus-prone mice (B6/*lpr*) that are genetically deficient in the receptor for IL-23 were protected from the massive lymphoproliferation, production of pathogenic anti-dsDNA antibodies, and the development of nephritis [5]. It is not known though whether blocking IL-23 will have an effect on the ongoing inflammatory response in lupus-prone mice. In this communication, we provide evidence that indeed treatment of lupus-prone mice with anti-IL-23 antibodies ameliorates nephritis through inhibition of the production of IL-17 by T cells. 

## 2. Materials and Methods

### 2.1. Animals

MRL/*lpr*/2J (MRL/*lpr*) were purchased from the Jackson Laboratories (Bar Harbor, ME, USA) and housed in the barrier animal facility of Beth Israel Deaconess Medical Center (BIDMC). The mice were injected with anti-IL-23p19 antibody (a gift from BD Biosciences) intraperitoneally three times a week. The Institutional Animal Care and Use Committee (IACUC) at BIDMC approved all animal-related procedures.

Urine was collected from each group of mice that were housed in metabolic cages overnight. The urine was analyzed for protein, blood, and white cells using the Multistix 10 SG reagent strips and the Clinitek Status Analyzer (Bayer Healthcare). All analyses were semiquantitative according to the manufacturer's instructions. Albumin and creatinine in the urine were measured using colorimetric assays according to the manufacturer's instructions (Albuwell M; The creatinine companion, Exocell).

### 2.2. Cell Culture and Activation

Cells were isolated from murine spleens and lymph nodes as previously described [6]. The cells were activated *in vitro *using a plate bound anti-CD3 (BD Biosciences) and CD28 antibody (BD Biosciences) at a concentration of 10 *μ*g/mL each. Murine IL-23 was added in the cultures as indicated. The anti-IL-23 antibodies and the IL-23 were added in the cell suspension immediately after the cells were added to the precoated wells.

### 2.3. ELISA

IL-17 was measured by ELISA (R&D Systems); IFN-*γ* was measured as part of a 7-cytokine flow cytometry-based array (Th1/Th17 cytokine bead array, BD Biosciences). Mouse anti-rat IgG antibodies were measured using an ELISA. Briefly, a 96-well plate was coated with rat IgG (BD Pharmingen) overnight and, after blocking and washing steps, was incubated with animal serum for 3 hours. Serial dilutions of mouse anti-rat IgG (Santa Cruz) were used as standards and goat IgG (Santa Cruz) as negative control. After several washings, the plate was incubated with goat anti-mouse IgG HRP conjugated detection antibody (Southern Biotech). After several washings, the HRP substrate was added and measurements were made using an ELISA reader. Mouse dsDNA serum levels were measured by ELISA (Alpha Diagnostic). Mouse IgG was measured by ELISA (Immunology Laboratories).

### 2.4. Statistical Analysis

The analyses were done using Graph Pad Prism 5.0. The unpaired two-tailed *t*-test was used. Statistical significance was defined as *P* < 0.05.

## 3. Results and Discussion

**Figure 1:**
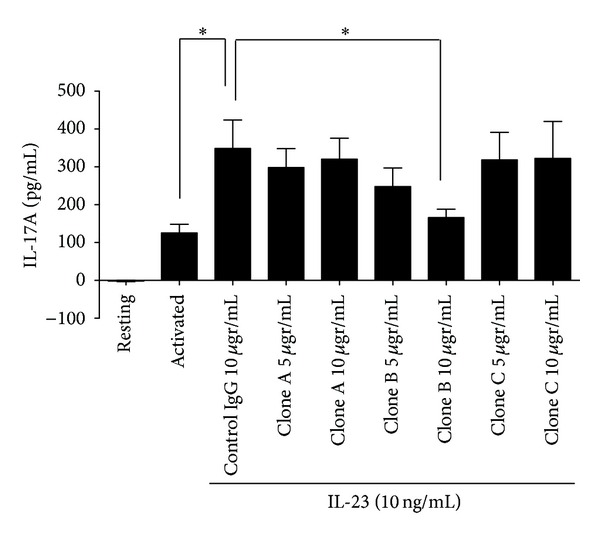
A monoclonal anti-IL-23p19 antibody limits the IL-23-induced production of IL-17 by MRL/*lpr *splenocytes. One million MRL/*lpr* splenocytes were activated *in vitro *in the presence of plate bound anti-CD3 and anti-CD28 antibodies (see Section 2). IL-23 was added in the medium as indicated at a concentration of 10 ng/mL. At 24 hours later, the concentration of IL-17 was measured in the supernatants using ELISA. Asterisk (∗) signifies statistical significance.

We initially screened *in vitro *three different monoclonal antibodies (hereby called clone A, B, and C) against IL-23 p19 subunit (BD Biosciences), which is unique for IL-23 and not shared with the related IL-12. All three clones were developed to bind *in vitro *to IL-23 but their relative biologic effectiveness in neutralizing IL-23 was unclear. MRL/*lpr* splenocytes were activated with plate-bound anti-CD3/CD28 antibodies *in vitro* in the presence or absence of interleukin-23. Different concentrations of anti-IL-23 antibodies (clones A, B, and C) or control IgG were added in the culture as indicated in Figure 1. The control rat IgG was used in order to control for nonspecific effect of immunoglobulin on splenocytes. The concentration of IL-17A was measured 24 hours later in the supernatants. As shown in Figure 1, anti-IL-23 treatment increased the production of IL-17 above and beyond anti-CD3/CD28 stimulation (*P* = 0.03). Of all the clones and the concentrations tested, only clone B at a concentration of 10 *μ*gr/mL was effective in blocking this IL-23-driven production of IL-17 (*P* = 0.05). 

**Figure 2:**
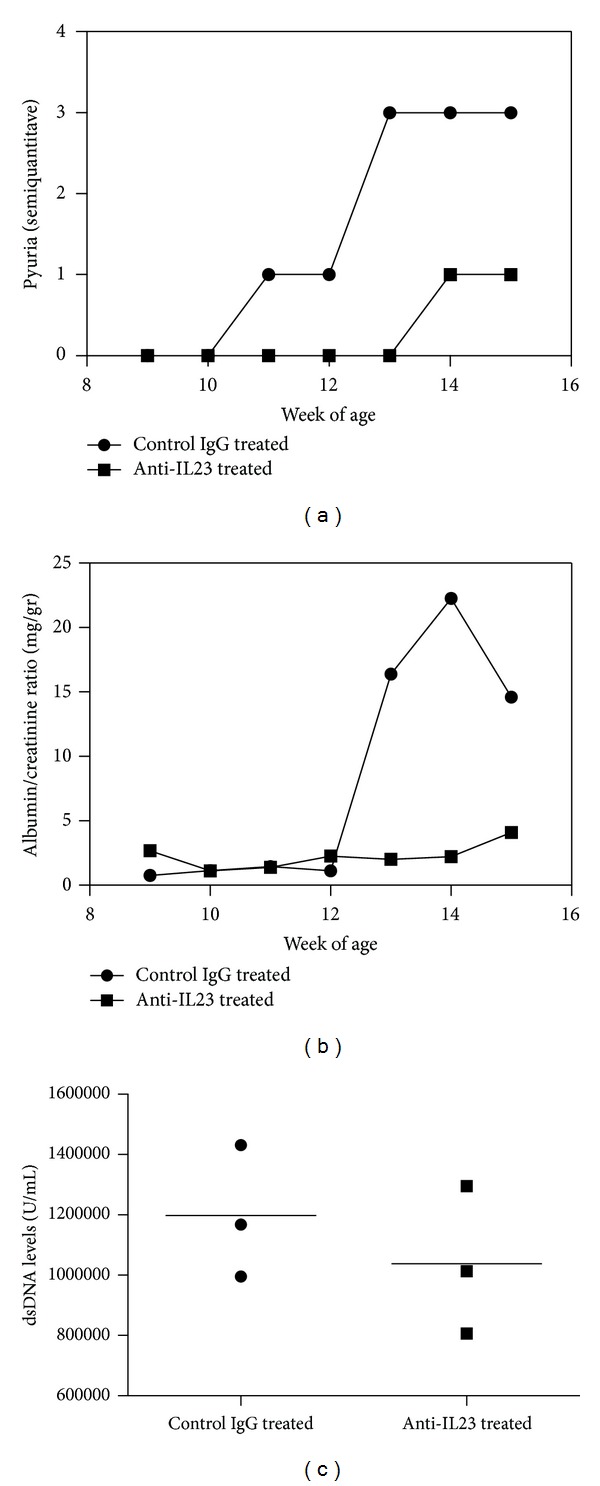
Six MRL/*lpr *mice were treated for the indicated time with 20 *μ*g of anti-IL-23p19 antibody given intraperitoneally three times weekly or control rat IgG. Urine was collected weekly. (a) The presence of leucocytes was assessed using a semiquantitative method. (b) Albumin and creatinine were measured using a colorimetric technique and the ratio is presented here. (c) dsDNA antibody levels were measured in the serum using a sandwich ELISA.

Subsequently, three 9-week MRL/*lpr* mice were injected with Clone B anti-IL-23p19 antibody at a dose of 20 micrograms per mouse three times a week intraperitoneally for six weeks. As controls, we used three mice of the same age and gender that were injected with the same amount of an unrelated monoclonal rat IgG antibody. At the initiation of the treatment, no mouse had an active urine sediment. Nevertheless, the mice had detectable anti-dsDNA antibodies in their serum suggesting that immunologic tolerance was already broken. As can be seen in Figure 2(a), only control treated mice developed pyuria. Similarly, the anti-IL-23 treated mice developed proteinuria at a lower level and at a later time-point than control treated mice (Figure 2(b)). Yet, at the end of the treatment, the size of spleens and number of cells in the spleen and lymph nodes were not different between the two groups. The levels of ds-DNA antibodies (Figure 2(c)) were similar between the groups. Moreover, total serum IgG was comparable between the two groups (control versus anti-IL-23 treated IgG (ng/mL): 576.6 ± 167.5 versus 702.1 ± 164.5, *P* = 0.4). These results suggested that this treatment had minimal effect on humoral immunity. Histologic examination of the kidneys at the end of the treatment disclosed no differences between the two groups. Mice from both groups had histologic findings of significant glomerulonephritis with cell proliferation, deposition of IgG and C3 in the glomerulus, and cell infiltration in the interstitium (data not shown). Additionally, none of the animals developed skin lesions during this trial.

**Figure 3:**
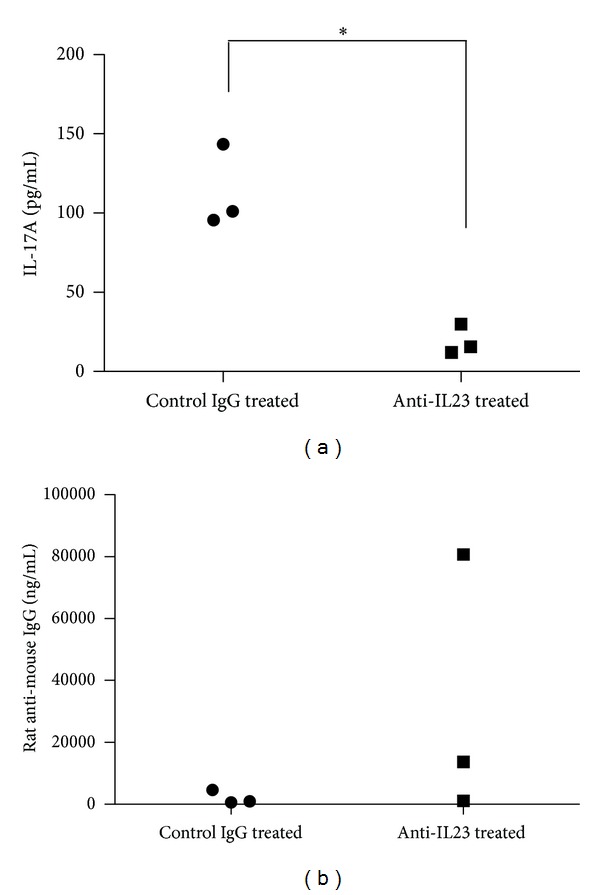
(a) At the end of the treatment, the MRL/*lpr *mice were sacrificed and the splenocytes were stimulated *in vitro* with anti-CD3/CD28 antibodies for 24 hours. IL-17A was measured in the supernatant by ELISA. (b) Serum levels of anti-rat IgG were measured by sandwich ELISA as described in Section 2.

At the end of the treatment, splenocytes were harvested and assayed *in vitro* for the production of cytokines. We found that splenocytes from the anti-IL-23 treated mice produced less IL-17A than control mice when stimulated *in vitro *(Figure 3(a)). There were no differences though between the two groups in the production of IFN-*γ*, IL-2, IL-6, and TNF-*α* (data not shown). These findings suggested that the anti-IL-23 treatment specifically decreased the production of IL-17 without significantly affecting Th1 cytokine production.

Given the fact that the injected antibody was rat anti-murine IL-23, we examined whether the mice developed anti-rat IgG antibodies. Indeed, we found that mostly the treated mice and less so the control mice developed murine anti-rat IgG (Figure 3(b)). Of note, the level of anti-rat antibodies did not correlate with the level of dsDNA antibodies in the serum of individual mice (data not shown). The presence of these antibodies may have negatively impacted the effect of anti-IL-23 treatment in these mice.

In this proof of concept study, we show for the first time that targeting IL-23 with a monoclonal antibody ameliorates nephritis in lupus-prone mice. This treatment was associated with a decrease in IL-17 production and no significant changes in Th1 activity, further emphasizing the pivotal role that IL-17 producing T cells are playing in lupus nephritis.

Another important finding was that the anti-IL-23 treatment was effective despite the fact that the treated mice had already established humoral autoimmunity at the beginning of the treatment. Moreover, production of total IgG and dsDNA autoantibodies by the treated mice did not differ from that of controls at the end of the study, suggesting that (a) the effect of this treatment was mediated primarily by its effect on T cells and (b) cellular responses are important in the pathogenesis of lupus nephritis independent of immune complexes/anti-dsDNA antibodies. It has to be pointed out though that the lack of significant decrease in anti-dsDNA antibody levels in the treated group may also be responsible for the modest effect of this treatment.

Besides the ineffectiveness of this antibody in decreasing anti-dsDNA levels, it also caused the production of anti-rat IgG antibodies at very high levels; this was also observed in mice treated with control rat antibody albeit at much lower levels. Human anti-drug antibodies have been well known to decrease the efficacy and cause infusion reactions of biologics such as anti-TNF antibodies used for rheumatoid arthritis [7]. Based on this well-known effect of biologics, we postulate that the development of mouse anti-rat antibodies may have significantly limited the effect of the anti-IL-23p19 antibody in abrogating nephritis. Future studies will be needed to fully characterize the usefulness of anti-IL-23 treatment in lupus, using murine anti-mouse antibodies that will not sensitize the mice and lead to inadvertent production of these anti-drug antibodies.

As mentioned above, we were not able to assess the role of IL-23 in the development of autoreactive B cells, as the mice already had high levels of anti-dsDNA at the start of the treatment. Our previous studies showed that anti-dsDNA levels were almost undetectable in lupus-prone mice that are IL-23 receptor deficient [5]. Contrary to that, elimination of IL-17 did not result in dsDNA level decrease in the Fc*γ*RIIb-deficient lupus-prone mouse [8]. Differences in the target (IL-23 versus IL-17), the mouse model, and the approach (genetic deletion versus inhibition) may account for these differences. Therefore, further studies will be needed to address the effect of IL-23 and Th17 cells in the development of humoral autoimmunity in lupus-prone mice.

In conclusion, these findings in lupus-prone mice call for further investigation of the role of IL-23 in SLE. As biologics targeting IL-23 become available [9], the precise effect of this cytokine in the generation, maintenance of autoimmunity, and the ensuing inflammatory damage of targetorgans will need to be elucidated in order to design novel therapeutic regimens for the treatment of SLE.
